# Cross‐sectional diagnostic accuracy study of self‐testing for proteinuria during hypertensive pregnancies: The UDIP study

**DOI:** 10.1111/1471-0528.17180

**Published:** 2022-05-12

**Authors:** Bethany Ellen Jakubowski, Richard Stevens, Hannah Wilson, Layla Lavallee, Lesley Brittain, Carole Crawford, James Hodgkinson, Lisa Hinton, Lucy Mackillop, Lucy C. Chappell, Richard J. McManus, Katherine Louise Tucker

**Affiliations:** ^1^ Nuffield Department of Primary Care Health Sciences University of Oxford Oxford UK; ^2^ Department of Women and Children's Health King's College London London UK; ^3^ Birmingham Women and Children's Hospital NHS Foundation Trust Oxford UK; ^4^ Institute of Applied Health Research, Murray Learning Centre, College of Medical and Dental Sciences University of Birmingham Birmingham UK; ^5^ THIS Institute, University of Cambridge, Clifford Allbutt Building, Cambridge Biomedical Campus Cambridge UK; ^6^ Nuffield Department of Women's and Reproductive Health University of Oxford Oxford UK

**Keywords:** diagnostic accuracy study, hypertension, pre‐eclampsia, pregnancy, proteinuria, self‐testing

## Abstract

**Objective:**

To determine the accuracy of self‐testing for proteinuria during pregnancy.

**Design:**

Diagnostic accuracy study.

**Setting:**

Antenatal clinics, maternity assessment units and inpatient wards at three hospital sites.

**Population or Sample:**

345 pregnant women.

**Methods:**

Pregnant women self‐tested in‐clinic for urinary protein using visually read dipsticks with samples then sent for laboratory estimation of the spot protein‐creatinine ratio (PCR) (primary reference test). Secondary index tests included testing by antenatal healthcare professionals and an automated colorimetric reader.

**Main outcome measures:**

Sensitivity, specificity, negative predictive value, positive predictive value and likelihood ratios were calculated for self‐testing (primary index test) along with healthcare professional and colorimetric testing compared to the primary reference test (PCR).

**Results:**

335/345 (97%) had sufficient data to be included in the analysis. Self‐testing had a sensitivity of 0.71 (95% confidence interval [CI] 0.62–0.79) and a specificity of 0.89 (95% CI 0.84–0.92) compared to PCR. Sensitivity and specificity of testing by healthcare professionals and the colorimetric reader were similar: sensitivity 0.73 (95% CI 0.64–0.80) and 0.78 (95% CI 0.69–0.85), respectively; specificity 0.88 (95% CI 0.82–0.92) and 0.83 (95% CI 0.78–0.88), respectively.

**Conclusion:**

Pregnant women can visually read a dipstick for urinary protein with similar accuracy to antenatal healthcare professionals. Automated colorimetric testing was not significantly different, in contrast to some previous studies. Self‐testing has the potential to form part of a self‐monitoring regime in pregnancy.

## Abstract 2

**Linked article**: This article is commented on by Wessel Ganzevoort, pp. 2149 in this issue. To view this mini commentary visit https://doi.org/10.1111/1471‐0528.17202

## 1 INTRODUCTION

Alongside blood pressure monitoring, dipstick proteinuria analysis is the most commonly performed antenatal screening test and is central to screening for pre‐eclampsia.^1^ It is often carried out by midwives or maternity support workers at routine antenatal visits, particularly for pregnant women at higher risk of pregnancy hypertension.^2^

The National Institute for Health and Care Excellence (NICE) and the International Society for the Study of Hypertension in Pregnancy (ISSHP) recommend the use of an automated colorimetric reagent strip (dipstick) reading device to screen for proteinuria in pregnant women with quantitative confirmation of positive tests using a protein or albumin‐creatinine ratio (PCR, ACR).^2^, ^3^ However, automated readers may not be regularly used: an online survey of 150 UK obstetricians found that the majority of respondents used visual assessment of a dipstick to screen for proteinuria initially.^4^ Automated readers are expensive and may not be feasible in all antenatal care settings, such as in primary care practices where many antenatal clinics take place.

Self‐testing, if accurate, could improve antenatal care and the detection of pre‐eclampsia as part of a self‐monitoring regime (blood pressure monitoring and self‐testing).^5^, ^6^ Self‐testing with dipsticks is inexpensive, convenient, easy to use, provides a rapid result and is common in diabetic care.^7^, ^8^ Pilot work in pregnancy suggests that women are willing and able to monitor their own urine alongside blood pressure monitoring with minimal training.^6^, ^9^ Linked qualitative work has shown that pregnant women and healthcare professionals are generally positive about such monitoring.^10^ However, there are currently few data on the accuracy of self‐testing for proteinuria in pregnancy.

The primary aim of this study was to determine the accuracy of proteinuria self‐testing by pregnant women with a diagnosis of chronic or gestational hypertension or pre‐eclampsia in comparison with a reference standard of laboratory protein: creatinine ratio (PCR) quantification. The secondary aim was to establish the accuracy of visual testing by a healthcare professional and by an automated colorimetric reader, compared to the same reference standard. The tertiary aim of this study was to compare the accuracy of these index tests to a secondary reference standard of laboratory albumin: creatinine ratio (ACR).

## 2 METHODS

### 2.1 Participants

Pregnant women aged 18–50 years with a diagnosis of gestational or chronic hypertension or pre‐eclampsia (defined by criteria set out by the International Society for Study of Hypertension in Pregnancy^11^) and ≥20 weeks’ gestation were recruited to the study at three hospital sites (Oxford University Hospitals, Guy’s and St Thomas’ Hospital, Birmingham Women and Children’s Hospital).

### 2.2 Procedure

Participants were recruited from a variety of settings within hospitals, antenatal clinics, maternity assessment units, and inpatient wards. Women interested in taking part were provided with information about the study prior to written informed consent. Participants were provided with simple instructions for protein testing (Figure S1), a urine sample pot with a funnel, and dipstick tests (ALBUSTIX reagent strips, Siemens, Surrey, UK) and undertook sample testing in the clinic or at the bedside on the same day they were recruited. Participants were asked to record their urinary proteinuria result on a case report form (CRF). Healthcare professionals, masked to the participant’s result, re‐tested the same urine sample using the same dipstick method. The same urine sample was then further tested using a dipstick test (URISTIX reagent strips; SIEMENS) in an automated colorimetric reader (Clinitek Status + Analyser; Siemens) by a member of the study team before being sent to the laboratory for PCR and ACR testing by a member of the laboratory team who were masked to other results. Urine samples were stored in line with current guidance and tested expediently in order to minimise sample degradation.^12^

### 2.3 Study outcomes and performance measures

The performance of dipstick tests was studied compared with laboratory PCR (primary reference test) and laboratory ACR (secondary index test). The primary index test was the dipstick result as read by the study participant, but secondary index tests were also considered: a healthcare professional visual read and an automated reader. Following Standards for Reporting of Diagnostic Accuracy Study (STARD) guidelines,^13^ performance measures were sensitivity, specificity, positive predictive value (PPV), negative predictive value (NPV) and likelihood ratios.

Index tests were categorised as negative, trace, 1+, 2+, 3+ and 4+, except when using the automated reader, which used categories negative, trace, 1+, 2+ and 3+. Categories ≥1+ were considered index test‐positive and categories ‘negative’ and ‘trace’ were considered index test‐negative.^2^, ^14^ Laboratory PCR test values of ≥30 mg/mmol and laboratory ACR test values of ≥8 mg/mmol were considered reference test‐positive, following recommendations from the NICE guidelines.^2^

### 2.4 Power calculation

It was anticipated that the index tests could have 80–90% sensitivity with respect to the primary index test,^6^ and that at least 100 complete cases of laboratory‐confirmed proteinuria would be required to estimate a sensitivity in this range with a standard error of no greater than 4% (95% CI‐width, ±1.96 × SE). To ensure sufficient cases, allowing for possible missing data, the case rate was monitored during recruitment, which was stopped when 110 cases with laboratory‐confirmed proteinuria had been recorded.

### 2.5 Statistical analysis

Data were analysed using STATA‐14. Descriptive statistics were reported for baseline characteristics. The sensitivity, specificity, PPV and NPV of the index tests were calculated against the primary reference test (PCR) and the secondary reference test (ACR) using the thresholds described above. Results for all dipstick thresholds (negative, trace, 1+, 2+, 3+, 4+) were shown on Receiver Operating Characteristic graphs. Likelihood ratios were calculated for all index tests. Samples missing index test or primary reference test data were excluded from the study.

## 3 RESULTS

**FIGURE 1:**
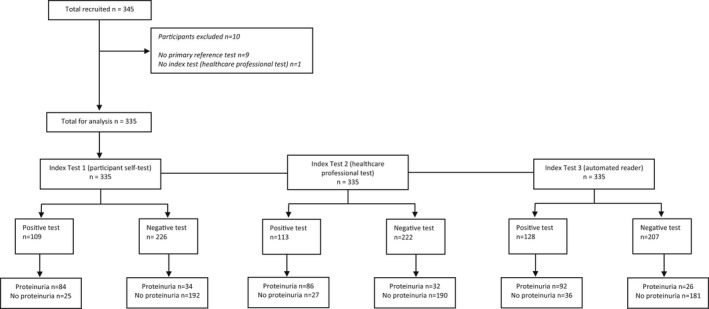
Standards of Reporting for Diagnostic Accuracy Studies (STARD) flow diagram for the UDIP study participants.

A total of 345 pregnant women completed the study (Figure 1). One exclusion was made due to a missing healthcare professional visual read result and nine exclusions were made due to unavailable laboratory PCR results, leaving 335 (97%) complete cases for the primary analysis. Eleven participants were missing only ACR results but were included in the primary analysis.

Thirteen (4%) participants were recruited but subsequently found not to meet the inclusion criteria: one was outside the maternal age range, and 12 were recruited below the gestational age specified in the inclusion criteria (at 12–19 weeks’ gestation). All 13 were included in the analysis using an ‘intention‐to‐treat’ principle, as it was considered that these protocol deviations were unlikely to bias the study.

Participating pregnant women had a median age of 33 years (IQR 29–37), with a median gestational age at recruitment of 34 weeks (IQR 27–36). The majority of women were in their first ongoing pregnancy (52%) and 118 (35%) had proteinuria quantified as ≥30 mg/mmol by the reference standard (PCR; Table 1).

**TABLE 1:** Demographic data of UDIP study participants

Demographics of study population (*n* = 335)		
	Median	IQR
Age	33	(29–37)
Gestational age at recruitment in weeks	34	(27–36)

	Frequency	%
Ethnicity		
Asian or Asian British	34	10.2
Black or black British	56	16.7
Mixed	12	3.6
White British	175	54.3
Other	58	15.2
First pregnancy	173	51.6
Prevalence of proteinuria	118	35.2

Abbreviation: IQR, interquartile range.

Participants’ self‐testing had a sensitivity of 0.71 (95% confidence interval [CI] 0.62–0.79) and a specificity of 0.89 (95% CI 0.84–0.92) compared with the primary reference test of PCR. The sensitivity of healthcare professionals and the automated reader respectively were 0.73 (95% CI 0.64–0.80) and 0.78 (95% CI 0.69–0.85), with specificity of 0.88 (95% CI 0.82–0.92) and 0.83 (95% CI 0.78–0.88), again compared with the primary reference test (Table 2). Similar results were found comparing index tests with the secondary reference test, ACR (Table S2). In a post‐hoc analysis suggested by a reviewer, kappa values were calculated for each index test compared with the primary reference test, PCR, and were 0.61 for self‐testing, 0.62 for healthcare professionals and 0.61 for the automated reader.

**TABLE 2:** Test performance for primary and secondary index tests against primary reference standard (laboratory PCR)

Threshold 1 + PCR (protein: Creatinine ratio)	Participants (Albustix) *n* = 335	Healthcare professionals (Albustix) *n* = 335	Automated reader (Clinitek status + Analyser, URISTIX) *n* = 335
Sensitivity *n*/*N*	0.71 (0.62–0.79) 84/118	0.73 (0.64–0.81) 86/118	0.78 (0.69–0.85) 92/118
Specificity *n*/*N*	0.89 (0.84–0.92) 192/217	0.88 (0.82–0.92) 190/217	0.83 (0.78–0.88) 181/217
Positive predictive value *n*/*N*	0.77 (0.68–0.85) 84/109	0.76 (0.67–0.84) 86/113	0.72 (0.63–0.80) 92/128
Negative predictive value *n*/*N*	0.85 (80.0–0.90) 192/226	0.86 (0.80–0.90) 190/222	0.87 (0.82–0.92) 181/207
Positive likelihood ratio	6.2	5.9	4.7
Negative likelihood ratio	0.3	0.3	0.3
False‐positive rate	0.11 24/217	0.12 26/217	0.17 35/217
False‐negative rate	0.29 34/118	0.27 32/118	0.22 26/118
Kappa value	0.61 (0.50–0.73)	0.62 (0.50–0.74)	0.61 (0.51–0.71)

Abbreviation: *n/N,* number/total number.

In a sensitivity analysis which excluded the 13 participants recruited in error, similar results were obtained (data not shown). Participants had a false‐negative rate of 0.29, with 22 of 34 having a laboratory PCR in the range of 30–50 mg/mmol. Healthcare professional testing and the automated reader had a false‐negative rate of 0.27 and 0.22, respectively, with 21 of 32 false negatives and 17 of 26 false negatives in the 30–50 mg/mmol range for PCR, respectively. The false‐positive rates were 0.11, 0.12 and 0.17, respectively.

**FIGURE 2:**
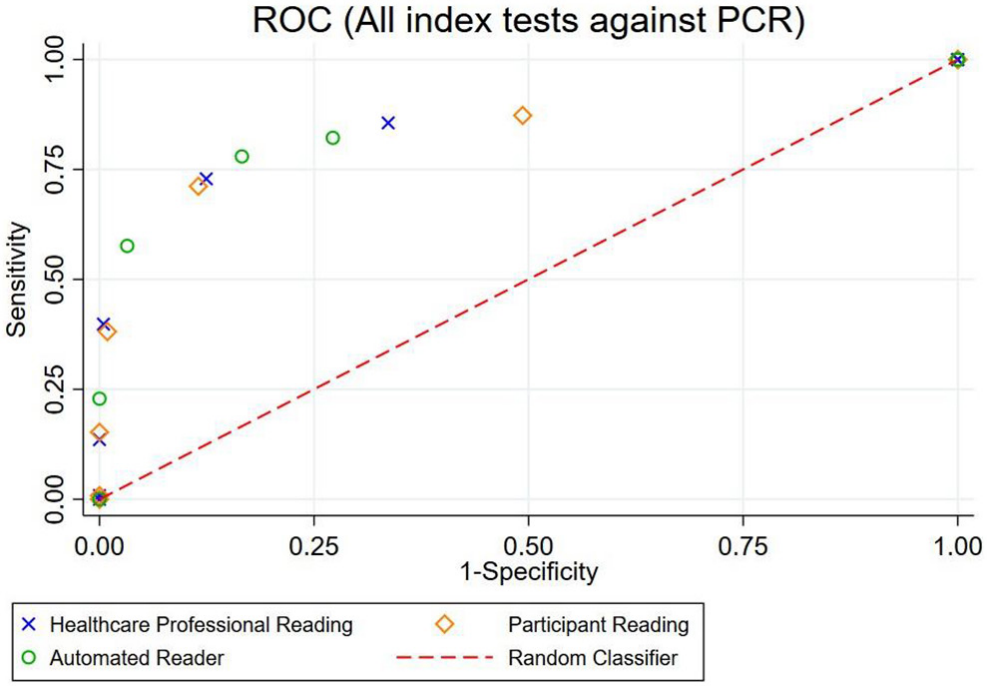
Sensitivity and specificity of three index tests, against the primary reference test (laboratory PCR), shown as a receiver‐operator characteristic plot

The test performance of the index tests compared with both primary and secondary reference tests is shown in a receiver‐operator characteristic (ROC) plot (Figure 2 and Figure S2). The ROC curves demonstrate comparable sensitivity across the index tests at multiple cut‐offs (negative, trace, 1+, 2+, 3+, 4+).

## 4 DISCUSSION

### 4.1 Main findings

Pregnant women self‐tested for proteinuria (≥1+) with similar accuracy to both healthcare professionals and a colorimetric reader. This held true whether laboratory PCR (≥30 mg/mmol) or ACR (≥8 mg/mmol) was used as the reference standard.

This research shows that dipstick tests have limitations regardless of who reads them, with around one in five false‐negative results (compared with a laboratory PCR); however, the majority of false negatives in this study were within the 30–50 mg/mmol range. NICE recommends repeat testing for any laboratory PCR result >30 mg/mmol if diagnostic uncertainty remains; for example, if there are no other clinical signs or symptoms of pre‐eclampsia (blood pressure ≥140 mmHg, maternal organ dysfunction, fetal growth restriction). This is motivated by the variation in protein excretion during the day and from day to day, and is intended to prevent a diagnosis of pre‐eclampsia on the basis of one raised PCR result.^2^ Previous evidence suggests that 30–50 mg/mmol is the range in which repeat laboratory testing would be beneficial.^14^, ^15^

Importantly, urinalysis is not done in isolation: women are asked about symptoms and have their blood pressure checked, and tests are repeated where any sign or symptom is of concern. The low proportion of false positives has fewer clinical implications, as this is a screening test where positive results are quantified with a laboratory test.

Dipstick urinalysis is cheap, convenient, easily repeatable, non‐invasive, and the most commonly used method of urinalysis in antenatal care. Repeat testing has been shown to increase the accuracy of other diagnostic screening tests, like blood glucose monitoring in diabetic care.^16^ Hypothetically, this would be the case with self‐testing for proteinuria, in order to reduce the number of false negatives. For example, a home self‐testing regime that focused on testing twice a week, as opposed to a one‐off test, is likely to increase the sensitivity, thus reducing the chance of a falsely reassuring negative test.^17^

### 4.2 Strengths and limitations

The strengths of this study include its size, with 335 women included in the analysis of this multi‐centre study representing a fully powered diagnostic accuracy study. The majority of participants (97%) completed the study and missing results were largely due to laboratory errors. To the authors’ knowledge, it is also the first study to compare accuracy across pregnant women, healthcare professionals and an automated reader, with two reference tests. Participants were recruited from a variety of hospital settings and included data from a range of pregnant women in the population most likely to be appropriate for self‐testing.^1^ Nearly half of the study population were from ethnic minority groups and this is important in the context of hypertension and pre‐eclampsia research. In the UK, black women with chronic hypertension are five times more likely than white women to experience adverse birth outcomes such as stillbirth, and Asian women with chronic hypertension are three times more like to experience adverse birth outcomes than white women.^18^

A further strength of this study was the use of PCR as the primary reference standard. Previously, 24‐hour urine collection was considered the appropriate comparator for proteinuria quantification^8^ but it has since been replaced in the UK with laboratory PCR testing. Recommended by NICE,^2^ PCR testing is more accurate,^15^ less time‐consuming and more convenient for women than 24‐hour urine collection.^19^, ^20^ The majority of studies examining point‐of‐care testing for proteinuria have used 24‐hour urine collection as a primary reference test, and those using PCR have not reported on self‐testing.^21^

A limitation to the study was that pregnant women performed a one‐off test in a clinic environment. Results may not perfectly reflect accuracy if carrying out repeated testing at home. Study participants did not have the responsibility to decide on the appropriate course of action following their test, as they would if self‐testing were included in a remote management regime. However, the results demonstrate that home self‐testing appears feasible and should be formally tested in future work with the aim of improving early detection of pre‐eclampsia in women with pregnancy hypertension.^22^

### 4.3 Interpretation

The results of this study showed that there was no clinically important difference between self‐testing by pregnant women and healthcare professionals. It also found no clinically important advantage to using an automated reader. This is discordant to previous findings which have reported a low sensitivity for healthcare professionals using visually assessed dipstick urinalysis (as low as 41%)^23^, ^25^ compared with automated readers. Of the studies that included an automated reader, the results varied, but they consistently reported a sensitivity for the automated reader <70%, using a range of dipsticks.^23^, ^25^ The ISSHP and NICE guidelines recommend using an automated reader^2^, ^3^ to reduce observer error and increase the sensitivity of the test.^26^

These findings align with pilot work using synthetic urine samples, which reported that healthcare professionals and pregnant women tested with similar accuracy, and the sensitivity and specificity of both index tests were comparable to those reported in this study (>70%).^6^ The differences between this study and previous studies could be due to the reference test; two studies that reported a low sensitivity for dipstick urinalysis used 24‐hour urine collection as the reference standard,^23^, ^24^ which is less accurate than a PCR test.^15^ Population differences could also account for discordant findings; one previously conducted study included non‐hypertensive pregnant women and therefore had a lower prevalence of proteinuria.^25^ Interpretation error may also account for the differences; this study used a single test dipstick, which only has one test to interpret, as opposed to previously used multi‐test dipsticks.

This study has reported findings consistent with previous studies on specificity (>80%).^23^, ^25^ The studies conducted previously^23^, ^25^ did not include self‐testing and the visual testing elements were performed by healthcare professionals or members of the study team.

The current method of in‐clinic urinalysis has limitations but is also easily repeatable, non‐invasive and usually used in combination with other tests. Urinalysis dipsticks are cheap and convenient, and therefore a self‐testing regime that incorporates either repeated home tests and/or a combination of home and clinic testing could support improved early detection of pre‐eclampsia. Dipstick testing remains the most accessible point of care test available in pregnancy and provides a rapid result compared with laboratory testing; moreover, it is a test that pregnant women can perform themselves. During the course of the COVID‐19 pandemic there has been an increase in remote antenatal care monitoring^4^, ^27^ for high‐risk pregnancies, repeat self‐testing combined with other self‐management activities, such as self‐monitoring blood pressure, has the potential to increase surveillance and information gathering in between antenatal visits, as well as increase confidence in remote care by including more elements of standard antenatal care checks.

Linked qualitative work suggests that self‐testing, potentially in a home setting, is acceptable to clinicians and pregnant women.^10^ Furthermore, automated readers are expensive and not suitable in all antenatal care settings. The comparable accuracy of these two methods suggests that a visually read dipstick is an appropriate alternative to an automated reader.

## 5 CONCLUSION

### 5.1 Clinical recommendations

Self‐testing appears a feasible alternative to an ‘in clinic’ dipstick test performed by a healthcare professional and suggests a home self‐testing regime could be considered. Self‐testing has comparable accuracy to the current methods of urinalysis used in antenatal care, and can be easily repeated to reduce the false‐negative rate.

### 5.2 Research recommendations

In light of the current COVID‐19 pandemic, finding feasible and safe alternatives to in‐person care has become a priority. This study demonstrates that pregnant women can accurately self‐test for proteinuria, but further research is needed on a home‐based self‐testing regime that focuses on the efficacy and accuracy of repeat self‐testing. Further health systems research is also needed to examine how self‐testing would work within existing care pathways and in combination with other self‐monitoring activates without substantially increasing the workload of healthcare professionals, and to evaluate how self‐testing could improve health equity and pregnant women’s experience of, and involvement in, antenatal care.

## CONFLICT OF INTEREST

RM has received BP monitors for research use from Omron and is working with them to develop a telemonitoring system for use in primary care. He receives no personal payment for such work. LM is a part‐time employee of Sensyne Health plc and holds shares in this company. The remaining authors have no disclosures. Completed disclosure of interest forms available to view online as supporting information.

## AUTHOR CONTRIBUTIONS

RM in collaboration with KT, LC, RS and LM gained the funding. The trial protocol was edited and agreed by all authors. Lead research midwives HW, LL and LB led the recruitment at sites. The trial was managed by KT and BJ. The analysis was conducted by BJ with support from RS. BJ, RS and KT wrote the first draft of the manuscript. All authors subsequently critically edited the manuscript. All authors have read and approved the final manuscript.

## ETHICAL APPROVAL

Ethical approval was provided by Yorkshire and the Humber ‐ Leeds East Research Ethics Committee (Reference: 18/YH/0208).

## PUBLIC AND PATIENT INVOLVEMENT

PPI representatives are on the steering committee for the programme grant that funded this work. Study progress and results were fed back to this committee at regular intervals, allowing for PPI contribution. Additionally, the application for the pilot work for this study was developed with PPI contributions.

## Supporting information


Figure S1

Figure S2

Table S1

Table S2

Caption

ICMJE

## Data Availability

The data that support the findings of this study are available from the corresponding author upon reasonable request.
