# Supplementary material for: Cross‐sectional diagnostic accuracy study of self‐testing for proteinuria during hypertensive pregnancies: The UDIP study
Source: BJOG. 2022 May 12;129(13):2142–8. doi: 10.1111/1471-0528.17180 (PMC9790635; doi:10.1111/1471-0528.17180)

Figure 3: UDIP Study Instructions

ID

U-DIP

Proteinuria Detection In Pregnancy (U-DIP)
Participant Visual Dipstick Reading Instructions

1. How many weeks pregnant are you? ________________
2. How old are you? __________________
3. Is this your first pregnancy? (circle) YES / NO
4. Have you been diagnosed with high blood pressure during this pregnancy? (circle)
   YES / NO
5. Please indicate your ethnic group

☐ Asian or Asian British	☐ White British
☐ Black or Black British	☐ White Irish
☐ Chinese	☐ Other (please specify _______
☐ Mixed	

6. Please provide the first four digits of the postcode of your home address (this will be used to look at the demographic distribution of participants)

___ ___ ___ ___

Using the test strip:

1. Dip the test pad of the strip into the sample and remove immediately.

2. Drag the edge of the strip against the container rim to remove excess liquid.

3. Compare each test pad to the colour blocks on the bottle label and note the result straight away on this form (ignore colour changes that occur after 2 minutes).

1

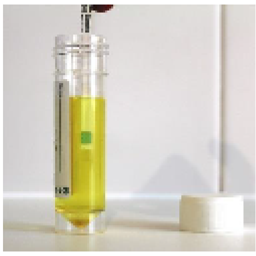

2

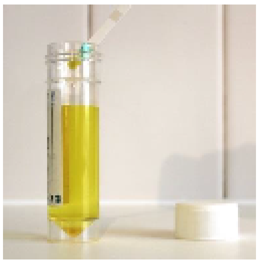

3

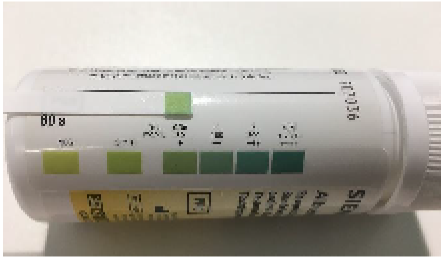

Supplement: Supplementary file 1 — Figure S1 [file BJO-129-2142-s004.pdf]
