# Supplementary figures and images for: Cross‐sectional diagnostic accuracy study of self‐testing for proteinuria during hypertensive pregnancies: The UDIP study
Source: BJOG. 2022 May 12;129(13):2142–8. doi: 10.1111/1471-0528.17180 (PMC9790635; doi:10.1111/1471-0528.17180)

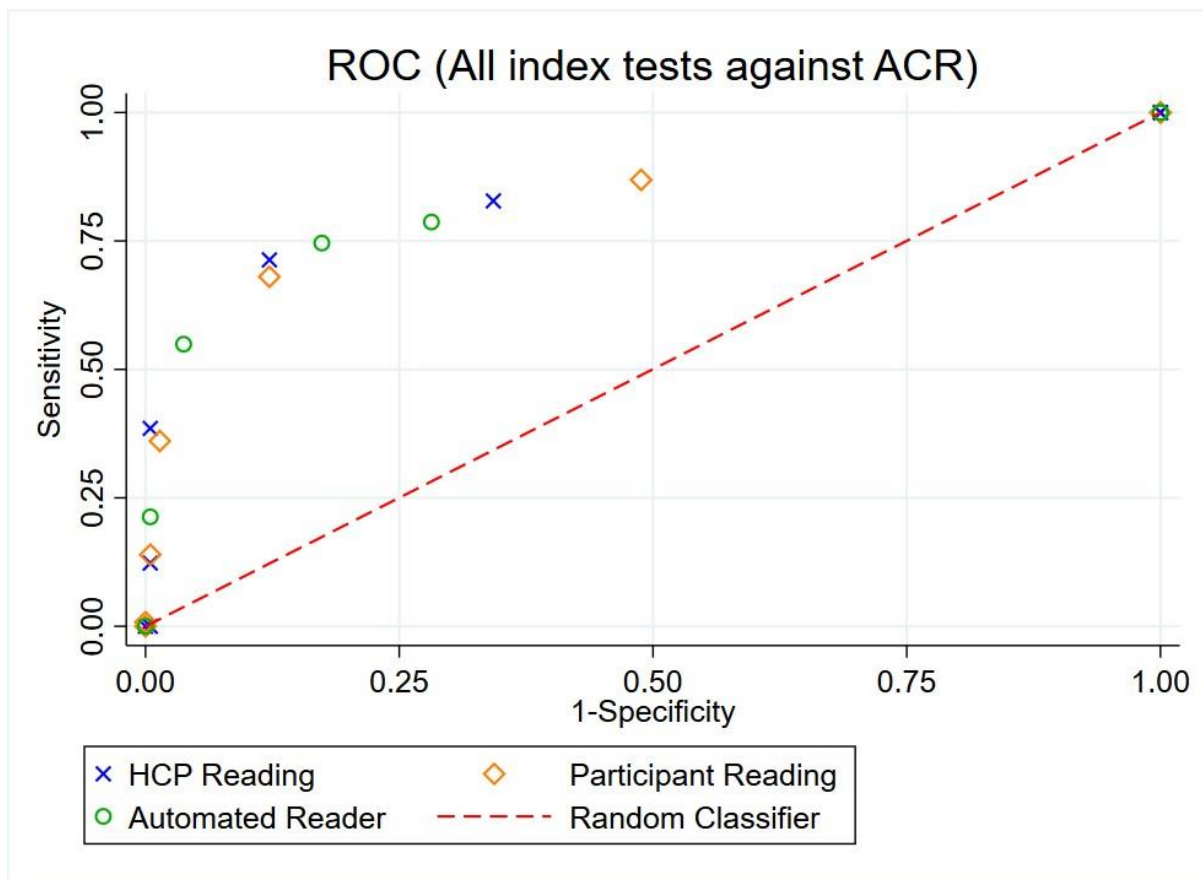

Supplement: Supplementary file 2 — Figure S2 [file BJO-129-2142-s003.pdf]
