# Supplementary material for: Cross‐sectional diagnostic accuracy study of self‐testing for proteinuria during hypertensive pregnancies: The UDIP study
Source: BJOG. 2022 May 12;129(13):2142–8. doi: 10.1111/1471-0528.17180 (PMC9790635; doi:10.1111/1471-0528.17180)
Supplement: Supplementary file 3 — Table S1 [file BJO-129-2142-s002.pdf]

Demographics of study population (n=335)		
	**Median**	**IQR**
**Age**	33	(29 - 37)
**Gestational age at recruitment in weeks**	34	(27 - 36)
	**Frequency**	**%**
**Ethnicity**		
Asian or Asian British	34	10.2
Black or Black British	56	16.7
Mixed	12	3.6
White British	175	54.3
Other	58	15.2
**First pregnancy**	173	51.6
**Prevalence of Proteinuria**	118	35.2
