# Supplementary material for: Cross‐sectional diagnostic accuracy study of self‐testing for proteinuria during hypertensive pregnancies: The UDIP study
Source: BJOG. 2022 May 12;129(13):2142–8. doi: 10.1111/1471-0528.17180 (PMC9790635; doi:10.1111/1471-0528.17180)
Supplement: Supplementary file 4 — Table S2 [file BJO-129-2142-s006.pdf]

*Threshold 1+ ACR (albumin:creatinine ratio)**	**Participants (Albustix)**	**Healthcare professionals (Albustix)**	**Automated Reader (Clinitek Status + Analyser, URISTIX)**
**Sensitivity**<br>**n/N**	0.68<br>(0.59 – 0.79)<br>83/122	0.71<br>(0.62 – 0.79)<br>87/122	0.75<br>(0.66 – 0.82)<br>91/122
**Specificity**<br>**n/N**	0.88<br>(0.83 – 0.92)<br>187/213	0.88<br>(0.83 – 0.92)<br>187/213	0.83<br>0.77 – 0.88)<br>176/213
**Positive predictive value**<br>**n/N**	0.76<br>(0.67 – 0.84)<br>83/109	0.77<br>(0.68 – 0.84)<br>87/113	0.71<br>(0.62 – 0.79)<br>91/128
**Negative predictive value**<br>**n/N**	0.83<br>0.77 – 0.87)<br>187/226	0.84<br>(0.79 – 0.89)<br>187/222	0.85<br>(0.79 – 0.90)<br>176/207
**Positive likelihood ratio**	5.6	5.8	4.3
**Negative likelihood ratio**	0.4	0.3	0.3
