# Supplementary material for: Cross‐sectional diagnostic accuracy study of self‐testing for proteinuria during hypertensive pregnancies: The UDIP study
Source: BJOG. 2022 May 12;129(13):2142–8. doi: 10.1111/1471-0528.17180 (PMC9790635; doi:10.1111/1471-0528.17180)
Supplement: Supplementary file 5 — Caption [file BJO-129-2142-s005.docx]

Figure S1: Study instructions provided to UDIP participants

Figure S2: Sensitivity and specificity of three index tests, against the secondary reference test (laboratory ACR), shown as a receiver-operator characteristic plot.

Table S1: Demographic data of UDIP study participants.

Table S2: Test performance for primary and secondary index tests against secondary reference standard (laboratory ACR).
